# Carcinosarcoma of the colon: report of a case with morphological, ultrastructural and molecular analysis

**DOI:** 10.1186/1471-2407-6-185

**Published:** 2006-07-12

**Authors:** Andrea Ambrosini-Spaltro, Valentina Vaira, Paola Braidotti, Marco PL Rovati, Stefano Ferrero, Silvano Bosari

**Affiliations:** 1 Pathology Unit, Department of Medicine, Surgery and Dentistry, A.O. San Paolo, via di Rudinì 8, 20142 Milan, Italy and IRCCS Foundation Policlinico Hospital, Mangiagalli and Regina Elena, University of Milan, Milan, Italy; 2 Abdominal and Thoracic Surgery Unit, Department of Medicine, Surgery and Dentistry, A.O. San Paolo, University of Milan, Milan, Italy

## Abstract

**Background:**

Carcinosarcoma of the colon is a rare histopathological entity with uncertain histogenesis, that shows both epithelial and mesenchymal malignant differentiation. Carcinosarcoma rarely affects the gastrointestinal tract and only few cases are reported in the colon. Herein we describe a carcinosarcoma of the ascending colon, with morphological, ultrastructural and molecular analysis.

**Case presentation:**

An 81-year-old man was hospitalised for asthenia, weight loss and iron-deficiency anaemia. The patient underwent colonoscopy and adenocarcinoma was diagnosed by endoscopic biopsy. A right hemicolectomy was performed and, during surgical operation, liver metastases were detected. Histological examination of the surgical specimen revealed areas of both carcinomatous and sarcomatous differentiation, completely separated by fibrous septae. The sarcomatous component exhibited areas of smooth muscle and osteoblastic differentiation, with focal osteoid material deposition. Molecular analysis conducted separately on the epithelial and mesenchymal components revealed the same *p53 *gene mutation (R282W in exon 8) and identical polymorphisms in *p53 *exon 4, in *EGFR *exons 20 and 21, and in *c-kit *exon 17. Microsatellite markers analysis revealed a common loss of heterozygosis on 18q. Overall, the data are consistent with a common origin of the two tumor components. The patient was treated with 8 cycles of oral capecitabine (1250 mg/m^2 ^twice a day for 14 days repeated every 28 days) and two years after surgery is alive with liver metastases.

**Conclusion:**

Carcinosarcoma of the colon is a rare tumour with both epithelial and sarcomatous components. Molecular analysis of the current case suggests the histogenesis from a common cell progenitor.

## Background

Carcinosarcoma is a rare histopathological entity, exhibiting both epithelial and mesenchymal malignant differentiation, with uncertain histogenesis. Carcinosarcoma has been described in various organs, although head and neck, and female urogenital system are the most frequent sites of occurrence [1]. In the gastrointestinal tract, carcinosarcoma arises predominantly in the oesophagus, in the stomach and in the biliary tract [2], whereas carcinosarcoma of the large intestine has been reported only rarely. This type of tumour generally displays an aggressive behaviour and poor prognosis. Herein we present a case of carcinosarcoma of the ascending colon.

## Case presentation

### Clinical features

An 81-year-old man with a past medical history of atherosclerotic heart disease, arterial hypertension, mitral insufficiency, bilateral carotid artery disease and early chronic renal failure was hospitalised for asthenia and weight loss. Laboratory investigation revealed iron-deficiency anaemia, with low haemoglobin (Hb) concentration (7.8 g/dl; normal range: 13–17 g/dL), median cellular volume (MCV) level (67.2 fl; normal range: 80–97 fl) and serum ferritin concentration (3.3 ng/mL; normal range: 30–400 ng/dL). Serum levels of carcinoembryonic antigen (CEA), carbohydrate antigen (CA) 19-9 and alphafoetoprotein (AFP) were within normal limits. Stool guaiac was positive for occult blood. Colonoscopy demonstrated an exophytic mass in the ascending colon and multiple diverticula in the entire colon, especially in the sigmoid region. Endoscopic biopsies were performed and at histological examination, adenocarcinoma was diagnosed. Chest x-ray was negative for metastatic disease. A week later a right hemicolectomy and regional lymph node dissection were performed. During surgery, liver metastases were detected. Computer tomography (CT) scan carried out after surgery identified 3 liver nodules in segment 4, in segment 8 and in segment 5, measuring 15 cm, 10 cm and 10 cm in their greatest dimension, respectively. The patient was subsequently treated with capecitabine at 1250 mg/m^2 ^orally twice a day for 14 days repeated every 28 days for 8 cycles. Capecitabine was chosen for adjuvant therapy in this case since it has been reported to be as effective as 5-fluorouracil but with milder side effects in stage III colon cancer [3]. No specific chemotherapeutic agents have been shown to be effective in carcinosarcoma [4]. Two years after surgical treatment, the patient is alive with liver metastases.

### Pathological features

#### Methods

Representative sections of the tumour and all lymph nodes isolated from the surrounding adipose tissue were fixed in 10% buffered neutral formalin, embedded in paraffin and routinely processed. From each block, 5 μm-thick sections were cut and stained with haematoxylin and eosin (H&E).

For immunohistochemical studies, the avidin-biotin peroxidase complex method was used with the following antibodies: cytokeratin 20 (CK20), desmin, vimentin, S-100, c-kit, CD34, sarcomeric actin, smooth muscle actin, osteonectin. The antibodies used are detailed in Table 1.

For ultrastructural examination, small samples were retrieved from paraffin-embedded material, deparaffinised in xylene, rehydrated in ethanol, post-fixed in osmium tetroxyde (OsO_4_), dehydrated and embedded in epoxy resin. Ultrathin sections were counterstained in uranile acetate and lead citrate and observed in a Jeol JEM 1010 (Tokyo, Japan) electron microscope operating at 80 kV.

For the molecular detection of somatic genetic alterations, the *p53 *gene (exons 4 through 9), the *c-kit *gene (exons 9, 11, 13 and 17) and the *EGFR *gene (kinase domain, exons 18 through 21) were evaluated by denaturing high-performance liquid chromatography (DHPLC) technique (The WAVE™ Nucleic Acid Fragment Analysis System, Transgenomic Inc., Nebraska, USA), followed by direct sequencing when required. *K-Ras *gene mutation status was assessed by direct sequencing. Distinct formalin-fixed paraffin-embedded material from both carcinomatous and sarcomatous components of the tumour was isolated by macrodissection. Genomic DNA was extracted by the phenol-chloroform method. Fifty ng of DNA were used for the specific amplification of all the exons listed above, as previously described [5-7]. After the polymerization reaction, all PCR (Polymerase Chain Reaction) products were visualised on a 2% agarose gel; 10 μl of the PCR product were denatured at 95°C and slowly cooled to room temperature to ensure equimolar homo- and heteroduplexes formation prior being loaded for DHPLC analysis. The running methods used are listed in Table 2. All samples that showed an elution profile different from a wild type control underwent direct sequencing by BigDye^® ^Terminator v1.1 Cycle Sequencing Kit chemistry, purified by DyeEx 2.0 Spin kit (Qiagen, Hilden, Germany) and analysed by capillary electrophoresis on Abi Prism 310 (Applied Biosystem, Foster City, California, USA). Sequences obtained were aligned with normal sequences and examined for detection of mutations. For allelic status testing, 10 microsatellite markers on chromosomal arms 9p, 11p, 13q, 17q and 18q were investigated (Rb, D9S171, D11S1336, D13S258, D13S317, D13S631, D17S855, D18S35, D18S51 and D18S585). Specific PCRs with fluorescent primers were conducted followed by loss of heterozygosis (LOH) analysis with GeneScan software (Applied Biosystem, Foster City, California, USA).

#### Results

The surgical specimen included a 4 cm long ileal segment, a 13.5 cm long colonic segment, and the caecal appendix. At the ileocaecal valve, on the colonic side, an exophytic, centrally ulcerated mass, measuring 7 cm in its greatest dimension, was documented. Twenty-two lymph nodes were isolated from the adipose tissue surrounding the bowel wall.

**Figure 1:**
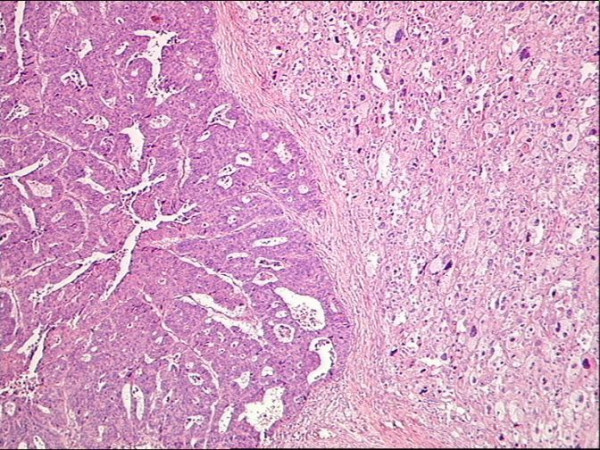
**Tumour appearance on H&E**. Carcinomatous component (left) and sarcomatous component (right) are completely separated by fibrous septae.

Microscopically, about 70% of the tumour displayed features of moderately differentiated adenocarcinoma, invading the subserosa (pT3). The carcinoma was composed of neoplastic epithelial cells, arranged in nests forming glandular structures with areas of central necrosis. Neoplastic epithelial cells exhibited rare mitotic figures, an increased nuclear/cytoplasmic ratio and prominent nucleoli. Approximately 30% of the tumour presented mesenchymal features. The sarcomatous component was completely separated from its carcinomatous counterpart by fibrous septae (Fig. 1). In the sarcomatous component, fascicular spindle cells admixed with bizarre large cells and focal osteoid-like matrix deposition were found. Fascicular spindle cells showed a moderately eosinophilic cytoplasm, with slightly enlarged nuclei. Large cells were markedly pleomorphic and exhibited enormously swollen nuclei with irregularly dispersed chromatin, focally surrounding areas of osteoid material deposition in the extracellular matrix. Metastasis of adenocarcinoma (N1) was found in 1 out of 22 lymph nodes detected. According to the TNM classification [8], the pathological stage was pT3N1.

**Figure 2:**
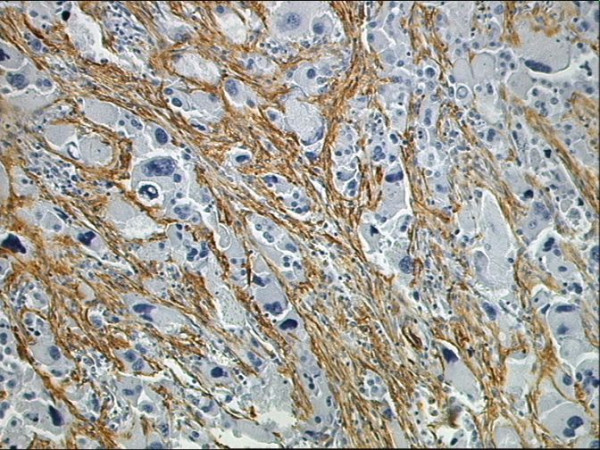
**Immunohistochemical analysis on the sarcomatous component (smooth muscle actin)**. Fascicular spindle cells of the sarcomatous component are immunoreactive for smooth muscle actin in their cytoplasm. Internal control is positive, as shown by blood vessels staining.

**Figure 3:**
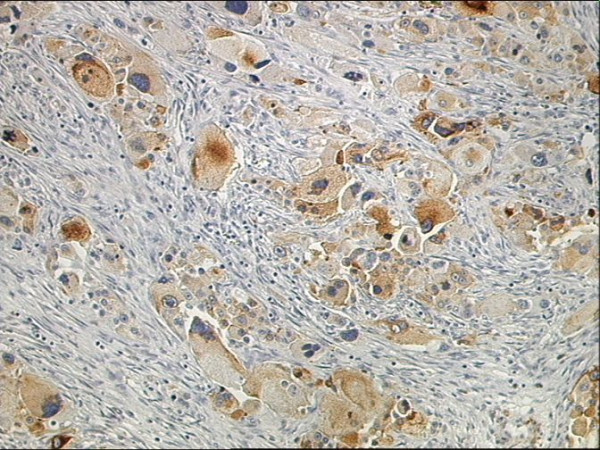
**Immunohistochemical analysis on the sarcomatous component (osteonectin)**. Bizarre large cells of the sarcomatous component are immunoreactive for osteonectin in their cytoplasm.

The epithelial component was immunoreactive for CK20 in approximately 30% of neoplastic cells, whereas it was negative for vimentin and osteonectin. In the mesenchymal component, spindle cells were diffusely immunoreactive for smooth muscle actin (Fig. 2) and partially positive for vimentin, while large cells were focally immunoreactive for osteonectin (Fig. 3). Both the epithelial component and the mesenchymal component were negative for S100, desmin, CD34, c-kit and sarcomeric actin.

**Figure 4:**
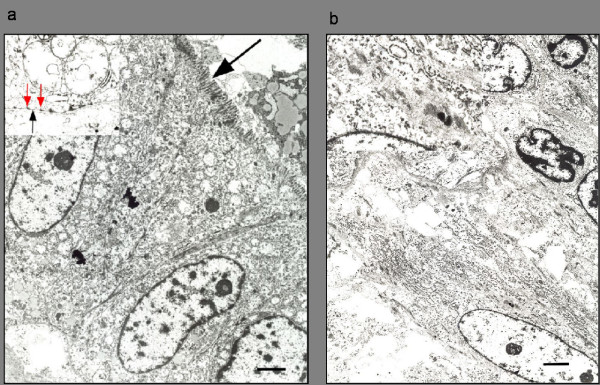
**Ultrastructural examination**: (a) Carcinomatous component: glandular epithelial structure with surface microvilla (arrow); in the inset: enlargement of the basement membrane (arrow) and hemidesmosomes (red arrows). (b) Sarcomatous component: spindle cells with actin cytoplasmic thin filaments and focal densities (enlargement in the inset). Scale bar: 2µm.

Ultrastructural examination was performed on both epithelial and mesenchymal areas of differentiation. Epithelial cells were organised in glandular structures encircled by basal membrane and showed typical surface microvilla and basal hemidesmosomes (Fig. 4A). Spindle cells exhibited myofibroblastic differentiation with peripheral cytoplasmic thin filaments and interspersed focal densities (Fig. 4B). There was no admixture of the two components.

**Figure 5:**
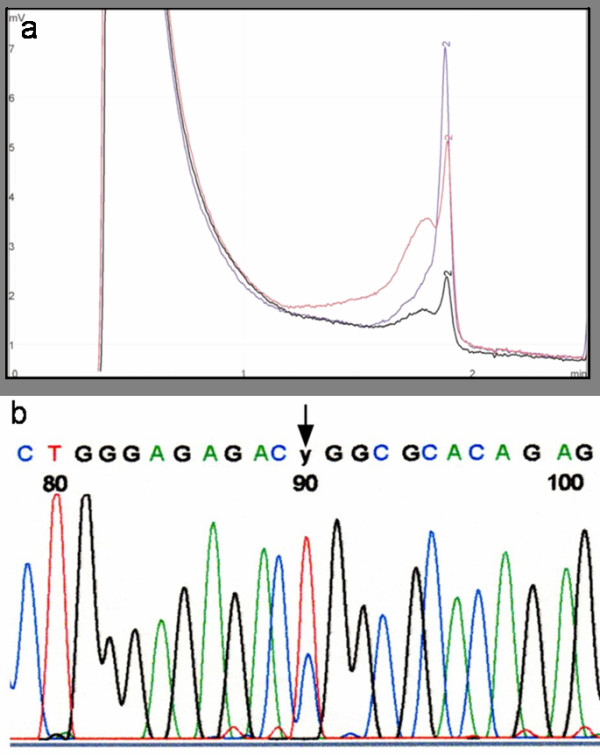
**DHPLC diagram and p53 sequencing analysis**. (a) DHPLC diagram. The carcinomatous component is represented in black while the sarcomatous component in red and the wild-type control sample in blue. The same genetic alteration in both tumour components is illustrated by the presence of an extra peak in the neoplastic specimens in comparison with the normal control. (b) Sequencing analysis showing p53 exon 8 mutation R282W (arrow).

**Figure 6:**
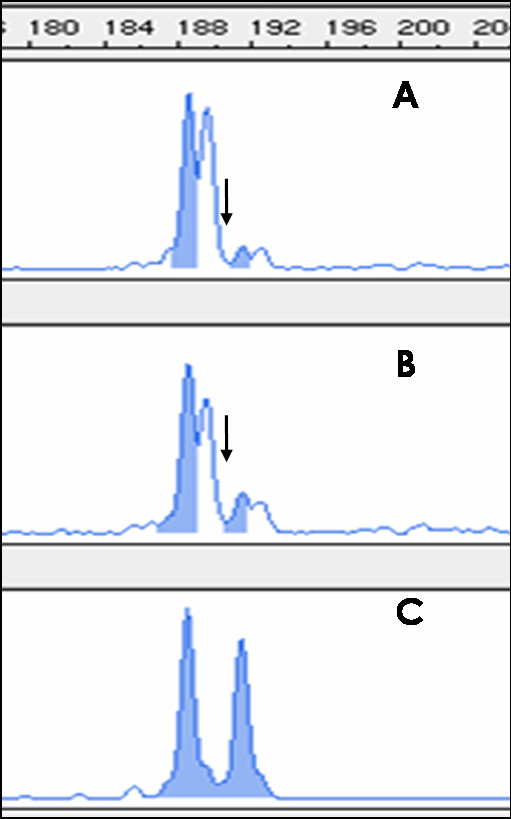
**STR analysis evidenced a loss of heterozygosity on 18q21**. Analysis of a representative marker of 18q21 (microsatellite D18S858) is shown. The carcinomatous (A) and sarcomatous (B) components shared an almost complete loss of the second allele (arrow) in comparison to the normal (C) counterpart.

The molecular screening performed to detect mutations revealed in both tumour components the same mutation R282W in exon 8 of the *p53 *gene (Figs. 5A, 5B). No mutation was found in the *k-ras *gene. Although no mutations could be detected, identical polymorphisms were identified in the carcinomatous as well as in the sarcomatous components in *p53 *exon 4, in *EGFR *exons 20 and 21, and in *c-kit *exon 17. The microsatellite analysis evidenced shared allelic retentions and loss among the epithelial and the mesenchymal neoplastic counteparts. Of the 10 STRs (Short Tandem Repeats) investigated, 7 were considered informative and four of them displayed an identical pattern comparing normal and the two tumoral components. An almost complete loss of the second allele was appreciated for D18S585, D18S35 and D18S51 markers (18q21) in both tumor' parts (Fig. 6).

### Discussion

Carcinosarcoma is widely considered a rare, highly aggressive tumour. Carcinosarcoma of the colon was first reported by Weidner et al in 1986 [9] and to the best of our knowledge, cases of colon carcinosarcomas and sarcomatoid carcinomas reported in literature are 17 [4,9][10-12][13-15][16-18][19-21][22-24], summarised in Table 3. Affected patients show an age between 41 and 84 years, with a median age of 66 years and a slight predilection for women. Lymph nodes and distant sites metastases disclose a predominance of the carcinomatous component. Shah et al reported even an increased epithelial component in the spleen metastasis, comparing with the primary colon site [19]. Only one case with a metastasis from the sarcomatous component was documented [18].

Herein we report a carcinosarcoma with distinct epithelial and mesenchymal components, at the morphological, immunohistochemical and ultrastructural levels. Moreover, the sarcomatous component was composed of cells reminiscent of smooth muscle differentiation and cells with osteoblastic appearance. Some authors classified as large bowel carcinosarcomas epithelial tumours with areas of sarcomatoid differentiation, weakly immunoreactive for cytokeratins and with no evidence of osteosarcomatous nor chondrosarcomatous differentation [12,13,20,21]. According to Aramendi et al., these cases are not properly carcinosarcomas, but should be considered sarcomatoid poorly differentiated carcinomas [23]. In the present case, the sarcomatous component completely lacked any epithelial signs of differentiation; furthermore, we noted areas of osteosarcomatous differentiation and osteoid material deposition. The topographic distribution was remarkable for the complete separation of the two components. Ultrastructural examination confirmed such distinct separation. Bertram et al. instead reported intermixed epithelial and mesenchymal components [14]. Other authors have classified as carcinosarcomas or sarcomatoid carcinomas tumours with sarcomatous features immunoreactive for cytokeratin and epithelial membrane antigen (EMA), but lacking carcinomatous component [4,15,16]. Shoji highlights the difficulty in making the correct diagnosis, since sarcomatous component closely resembles sarcoma, except for cytokeratin-immunoreactivity [16].

Treatment, as underlined by Bertram et al., should follow guidelines for common colon adenocarcinomas [14] and the poor prognosis associated with this tumour should require a strict follow-up. Remarkably, the patient reported here is still alive, two years after surgical resection of the primary tumour, despite the presence of liver metastases. Therefore we believe that aggressive therapy may be indicated in these tumours.

The histogenesis of carcinosarcoma is still controversial. Morphologically, the presence of distinct carcinomatous and sarcomatous components suggests a different origin (multiclonal hypothesis). The molecular analysis performed in the current case supports the hypothesis of a common cell precursor (monoclonal hypothesis), since the same mutation R282W in exon 8 of the *p53 *gene as well as the same allelic status, with the loss of 18q21, were identified in both carcinomatous and sarcomatous components.

Studies conducted on carcinosarcomas of nasopharynx [25], uterus [26,27] and breast [28], documented a large overlap of cytogenetic and molecular alterations in the two tumour components. Furthermore, Van Rees et al. [29] described an uncommon case of adenosquamous carcinoma raised in a Barrett esophagus where the two malignant components showed loss of the same allele at all informative chromosomal markers tested as well as the same missense mutation in the p53 tumor-suppressor gene. All these findings are consistent with our results suggesting the hypothesis of a common progenitor of both epithelial and mesenchymal components. Therefore morphological divergence, even at the extreme levels displayed by carcinosarcomas, necessarily appears late in tumor progression, well after the initial hits that cause cancer.

## Conclusion

Herein we describe a case of carcinosarcoma of the colon, with epithelial and mesenchymal components, completely different and separate at the morphological, immunohistochemical and ultrastructural levels. Molecular analysis revealed the same mutation R282W in exon 8 of the *p53 *gene and identical LOH for D18S585, D18S35 and D18S51 in both carcinomatous and sarcomatous components, supporting the hypothesis of a common cell progenitor.

## Competing interests

The author(s) declare that they have no competing interests.

## Authors' contributions

AAS revised the literature and drafted the manuscript. VV conducted the molecular analysis and participated in writing the manuscript. PB carried out the ultrastructural examination. MR performed the surgical operation and clinical follow-up. SF and SB devised the study and revised the manuscript. All the authors read and approved the final manuscript.

## Pre-publication history

The pre-publication history for this paper can be accessed here:



## Figures and Tables

**Table 1:** List of antibodies used

**Antiserum**	**Source**	**Dilution**	**Clone**
Citokeratin 20	Novocastra	40	K_s_20.8
Desmin	Dako	100	D33
Vimentin	Dako	1000	V9
S100	Dako	10000	Polyclonal
c-kit, CD 117	Dako	200	Polyclonal
CD 34	Novocastra	200	QBEnd/10
Sarcomeric Actin	Dako	200	Alpha-Sr-1
Smooth Muscle Actin	Biogenex	1000	1A4
Osteonectin	Biodesign	50000	N50

**Table 2:** Running methods and temperature used for DHPLC mutation detection

**Gene-Exon (bp)**	**Starting Gradient (%B)**	**Temperature (°C)**
*p53*-4	58.2	60.9/62.5
*p53*-5I	50	64.4
*p53*-5II	52.9	65.1
*p53*-6	53.1	61.5
*p53*-7	53.5	61.7/63.4/64.3
*p53*-8	54.6	59.8/62.8
*p53*-9	50.5	58.6/59.6

*c-kit*-9	56.5	55.3/58.5/59.5
*c-kit*-11	51.2	57.7/58.7
*c-kit*-13	55.4	56.7/58.7/59.3
*c-kit*-17	54.2	56.5/58

*EGFR*-18 (400)	57	60.9/62.9
*EGFR*-19 (372)	56.5	60.1
*EGFR*-20 (409)	57.2	61.2/63.4
*EGFR*-21 (415)	57.3	60.8

**Table 3:** Cases of carcinosarcoma reported in literature

**Case**	**Author, year**	**Age (years)**	**Sex**	**Site**	**Distant Metastasis**	**Outcome**
1	Weidner, 1986 [9]	73	M	Sigmoid	F	DOD 4 years
2	Staroz F, 1995 [10]	64	M	Descending	F	DOD some months later
3	Roncaroli, 1995 [11]	71	F	Rectum	F	DOD 6 months
4	Isimbaldi, 1996 [12]	86	F	Ascending	None	NED 2 years
5	Gentile, 1997 [13]	40	F	Caecum	I	DOD 2 months
6	Bertram, 1997 [14]	79	F	Ascending	I	DOD 5 months
7	Serio, 1997 [15]	69	F	Descending	None	NED 6 months
8	Shoji, 1998 [16]	78	M	Descending	None	NED 16 months
9	Nakao, 1998 [17]	60	F	Transverse	None	NED 14 months
10	Takeyoshi, 2000 [18]	82	M	Rectum	F	DOD 6 months
11	Shah, 2001 [19]	57	F	Rectosigmoid	I	DOD 5 months
12	Kim, 2001 [20]	41	F	Sigmoid	None	DOD 4 months
13	Di Vizio, 2001 [21]	56	F	Descending	F	DOD 21 months
14	Ishida, 2003 [22]	80	F	Rectosigmoid	F	DOD 6 months
15	Aramendi, 2003 [23]	84	M	Splenic flexure	None	ARF after 4 hours
16	Macaigne, 2004 [24]	67	F	Sigmoid	F	DOD 2 months
17	Kim, 2005 [4]	71	M	Ascending	I	NS
18	Present Case	81	M	Ascending	I	AWD 2 years

I metastasis detected at initial presentation, F metastasis detected during follow-up, DOD died of disease, AWD alive with disease, NED no evidence of disease, ARF acute renal failure, NS not specified
